# Not Always a Wise Procedure

**Published:** 1897-05

**Authors:** 


### NOT ALWAYS A WISE PROCEDURE.

THE rapidly changing conditions of the times, calling to the aid of cities, towns, and boroughs, the assistance and aid of milk- and meat-inspectors, associated with the progressive movements of health and sanitary boards, have found, in many instances, that this field of work had not been properly cultivated, and a lack of education of the public has not created a discerning recognition of those best equipped to fill such important positions. By training, education, and association there is no one so well prepared to discharge these duties as the educated veterinarian. No layman, no butcher, no cattle-breeder or owner can, under any circumstances, discharge properly the duties of such positions; neither is the mere graduate of human medicine, without long experience and a knowledge of the domesticated animals, gained by observation and training, so well equipped to fill such posts. But it is of the layman we wish to speak more especially, and to say to our colleagues, when seeking these positions, that they must not forget that their services are much more valuable than those of a layman. It is trained skill of a high order they are offering, and, in the race for place or in the zeal to dislodge an unqualified person from such places, the veterinarian must not forget the greater value of his services and that a more fitting compensation should follow. To offer scientific, technical skill at little cost, or the throwing out of bargain-counter inducements is not always a wise procedure, and proves often a two-edged sword by first affording an inadequate compensation for work which should be properly and conscientiously performed; and secondly, fixes a low rate of compensation for similar services in other cities and towns, thus injuring all who may be desirous of elevating the profession.

---

THE Wyoming Valley Veterinary Medical Association passed strong resolutions and charged their officers with the duty of protesting against the proposed reopening of the Act of 1889, governing the registration of non-graduates in Pennsylvania. This association, in conjunction with members of the State Veterinary Examining Board, will press to an ultimate decision several violations of the laws governing the practice of veterinary science in Lackawanna County.

---

THE death of so many cattle and sheep on the Western ranges from the severe cold of the past winter has called forth a stinging rebuke from Prof. Thomas Shaw, of the Minnesota Agricultural College, to the men whose inhumanity imperilled the lives, not to speak of the suffering, of these animals, by placing them in such dangers, without making provision for either food or shelter. The humane societies of our land are strongly called upon to take action concerning this inhuman exposure of animals to such uncalled-for danger and suffering.

---

THE several proposed legislative acts of interest to the veterinarians of Pennsylvania are making progress. The bill to reopen registrations has not yet been reported from the committee to which it was referred. The bill to prevent the introduction of cattle for breeding or dairy purposes, except with certificate of freedom from tuberculosis or submission to the tuberculin-test, is well advanced on the calendar, but needs the continued advocacy and support of all those interested in these branches of animal industry. The bill to appropriate fifty thousand dollars for the purposes of investigation of the infectious and contagious diseases of live-stock, so as to arrive at more definite plans of dealing with them, is in the hands of the Appropriation Committee. This bill has won for itself the earnest support of the press, the farmers and dairymen, the veterinary and medical profession, as well as all boards of health and sanitary bodies.

---

GOVERNOR TANNER, of Illinois, shows little indication of any high conception of the needs of the times, and, in his recognition of a non-graduate for State Veterinarian, gives small encouragement to the movement demanding higher veterinary education in his State by the establishment of a Veterinary Department at the State University.

---

THE political shake-up in the Kansas Agricultural College, involving the retirement of some fifteen of the trustees and faculty, includes among the number N. S. Mayo, Professor of Physiology and Veterinary Science. Such are the dangers of State Universities which are semi-political in character, and where changes of administration in State government are often followed by changes in the State educational institutions, from which it will take years to recover. Kansas seems to be a cyclonic State in more ways than one.

---

VERY important plans and experimental trials for better information as to the methods of propagation and spread of tuberculosis are being planned by the State Veterinary Sanitary Board of Pennsylvania. These tests, bearing upon the practical and monetary measures of control, will be of the utmost value in dealing with this scourge, limiting its extension and determining the barriers necessary to control its ravages.

---

THE Bay State Legislature at its last session voted the sum of $250,000 for the work of the Cattle Commission in stamping out tuberculosis and other infectious and contagious diseases of live-stock within its borders. The changes in the constitution of the

Commission brought to its control and direction two members of our profession whose views, privately and publicly, were antagonistic to those members of our calling who had matured plans for dealing with the scourge of tuberculosis. The whole country waits with impatience to learn of the plans of those now in control; they appreciate the value of the great power in the Commission's hands; they are fearful of the loss of this power if some definite plan and work are not consummated; they realize better, perhaps, the result in other States where smaller sums of money were appropriated and used up in work without method or plan, and they are more than anxious that this work shall not be wiped out or placed in the hands of laymen because our profession is drifting about without plans or distinct purposes in the veterinary sanitary field of work. Much is known about human and bovine tuberculosis, but there is a great deal more to learn before economical plans for its control can be adopted, and these can only be determined by distinct and well-directed experimental work in now well-indicated directions. The country waits and the profession is dependent, in a measure, upon the lead in Massachusetts. The dangers are far-reaching, the burdens are heavy upon those who are carrying them, and we do not want to hear the criticism that such an intelligent body are, Micawber-like, waiting for "something to turn up." Let us hear of your turning up something; let us know of your plans, that others may join you in the common end of it all—relief to all concerned.

